# Relatedness between the two-component lantibiotics lacticin 3147 and staphylococcin C55 based on structure, genetics and biological activity

**DOI:** 10.1186/1471-2180-7-24

**Published:** 2007-04-02

**Authors:** Eileen B O'Connor, Paul D Cotter, Paula O'Connor, Orla O'Sullivan, John R Tagg, R Paul Ross, Colin Hill

**Affiliations:** 1 Moorepark Food Research Centre, Teagasc, Fermoy, Co. Cork, Ireland; 2 Department of Microbiology, National University of Ireland, Cork, Ireland; 3 Alimentary Pharmabiotic Centre, Cork, Ireland; 4 Department of Microbiology, University of Otago, Dunedin, New Zealand

## Abstract

**Background:**

Two component lantibiotics, such as the plasmid-encoded lacticin 3147 produced by *Lactococcus lactis *DPC3147 and staphylococcin C55 produced by *Staphylococcus aureus *C55, represent an emerging subgroup of bacteriocins. These two bacteriocins are particularly closely related, exhibiting 86% (LtnA1 and C55α) and 55% (LtnA2 and C55β) identity in their component peptides. The aim of this study was to investigate, for the first time for any two component bacteriocins, the significance of the relatedness between these two systems.

**Results:**

So close is this relatedness that the hybrid peptide pairs LtnA1:C55β and C55α:LtnA2 were found to have activities in the single nanomolar range, comparing well with the native pairings. To determine whether this flexibility extended to the associated post-translational modification/processing machinery, the staphylococcin C55 structural genes were directly substituted for their lacticin 3147 counterparts in the *ltn *operon on the large conjugative lactococcal plasmid pMRC01. It was established that the lacticin LtnA1 post-translational and processing machinery could produce functionally active C55α, but not C55β. In order to investigate in closer detail the significance of the differences between LtnA1 and C55α, three residues in LtnA1 were replaced with the equivalent residues in C55α. Surprisingly, one such mutant LtnA1-Leu21Ala was not produced. This may be significant given the positioning of this residue in a putative lipid II binding loop.

**Conclusion:**

It is apparent, despite sharing striking similarities in terms of structure and activity, that these two complex bacteriocins display some highly dedicated features particular to either system.

## Background

Lantibiotics are gene-encoded, ribosomally-synthesized antimicrobial peptides which are distinguishable by the presence of unusual amino acids including lanthionine (Lan), β-methyl-lanthionine (meLan) and a number of dehydrated amino acids (for comprehensive reviews see [1-4]). These unusual amino acids are formed as a result of post-translational modifications of precursor peptides; for example, serine and threonine residues are enzymatically dehydrated to give dehydroalanine and dehydrobutyrine, respectively. A cysteine residue can then react with one of the newly formed unsaturated sites in what is essentially a 1,4-Michael addition reaction to form the characteristic thioether amino acids Lan and meLan. Lantibiotics are synthesized as precursor peptides containing N terminal extensions or leader peptides which are removed during export through a dedicated bacteriocin transport system leading to the active mature peptide.

Lantibiotics are currently classified into eleven subgroups based on alignments of the unmodified structural peptides (for the most recent classification scheme see [4]). Two of these groups contain the individual components of a number of two component lantibiotics i.e. those lantibiotics which display enhanced bactericidal effects due to the complementary activity of two peptides. To date, seven two component lantibiotics have been identified and include lacticin 3147 [5], staphylococcin C55 [6], plantaricin W [7], cytolysin [8], haloduracin [9], Smb [10] and BHT-A [11]. Of these, cytolysin is clearly the most distant relative based on homologies and biological activity, while lacticin 3147 and staphylococcin C55 peptides are particularly closely related. Staphylococcin C55 is produced by *Staphylococcus aureus *C55, the adopted prototype of phage II bacteriocin producers (7) and indeed, is widely produced by this group of *S. aureus *strains [12]. Its genetic determinants are located on a 32 kb plasmid in the strain *S. aureus *C55 [6] but have also been identified on a 37 kb plasmid in *S. aureus *U0007 (Warren *et al*., 1975) and on a 38 kb pETB plasmid from the clinical isolate *S. aureus *TY4 [13]. Interestingly, in all cases, the bacteriocin structural genes are closely associated with an exfoliative toxin B determinant, an exotoxin associated with skin infections in humans [14]. The structural peptides, staphylococcin C55α and C55β of molecular masses 3339 and 2993, respectively, are both required in equimolar amounts to act synergistically to give an antimicrobial effect against *S. aureus*, *Micrococcus luteus *but not *S. epidermidis *strains [6]. Mode of action studies indicated that cell death was due to pore formation in the cytoplasmic membrane and widespread inhibition of macromolecular biosynthesis following exposure to the partially purified material (Dajani *et al*., 1973).

In contrast, lacticin 3147 is produced by a food-grade *L. lactis *subsp. *lactis *DPC3147 strain and encoded on a 60.2 kb conjugative plasmid pMRC01 which encodes the genetic determinants for production and immunity [15]. The two structural peptides of lacticin 3147 are LtnA1 and LtnA2 with molecular masses of 3306 and 2847, respectively. The optimal ratio of the two lacticin peptides has recently been established, suggesting a peptide stoichiometry of 1:1, effective at nanomolar concentrations (7 nM) [16]. Lacticin LtnA1 does exhibit independent inhibitory activity which is greatly enhanced by the presence of the LtnA2 peptide. Sequential addition studies show that the LtnA1 peptide must be added before LtnA2 to observe inhibitory activity [16]. This activity, which occurs at nanomolar concentrations for the lacticin peptides, suggests the involvement of a docking molecule speculated to be lipid II as is the case with nisin [17]. In addition, lipid II has been identified as the target molecule for a number of other antimicrobial peptides including mersacidin and actagardin [18-20]. Interestingly, the 3D structure of lacticin 3147 has recently been elucidated [21] and reveals a specific lanthionine-bridging pattern in LtnA1 closely resembling that of the lipid II-binding globular lantibiotic mersacidin which acts by inhibition of cell wall biosynthesis. In contrast, the LtnA2 peptide has a more elongated linear structure potentially capable of pore formation. It is speculated that the lacticin peptides display a dual mode of action similar to nisin, however in the case of lacticin 3147 both functions are assigned to separate peptides [16]. In essence, it is predicted that LtnA1 binds to lipid II in a similar way to mersacidin, thereby inhibiting cell wall biosynthesis through the prevention of transglycosylation. The second function of pore formation and rapid efflux of K+ and phosphate ions [22] is assigned to the linear more hydrophobic peptide LtnA2. Weidemann *et al. *[43] recently provided some experimental evidence that agreed with this speculation.

The focus of this communication was to investigate the similarities and differences between lacticin 3147 and staphylococcin C55. Primary sequence analysis of the prepeptides suggests that lacticin A1 and staphylococcin C55α resemble natural variants with a difference of only 4 amino acids between the two peptides, while the complementary peptides share less identity (55%). We demonstrate that purified peptides of the two bacteriocins can cross complement with negligible losses in activity, and that the post translational machinery of LtnA1 can produce a viable C55α peptide. Furthermore substitution of leucine 21 of LtnA1, located within a proposed lipid II binding pocket, for the corresponding alanine residue in C55α counterpart, was not produced.

## Results and Discussion

### Bioinformatic analysis

**Figure 1:**
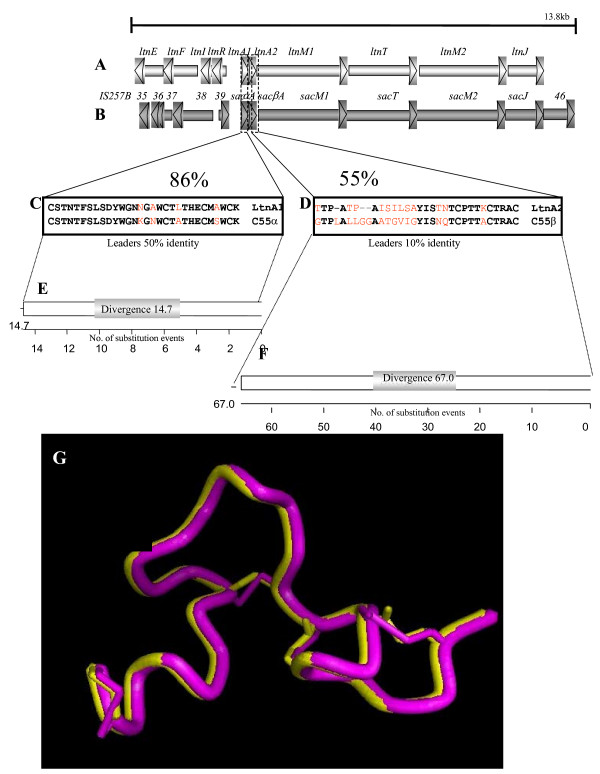
Genetic organisation of the gene clusters of the closely related lantibiotics lacticin 3147 (A) and staphylococcin C55 (B). C depicts a comparison of the amino acid sequence of the propeptide of one of the structural components of lacticin 3147 (LtnA1) and staphylococcin C55 (C55α). D depicts a comparison of the amino acid sequence of the propeptide of one of the structural components of lacticin 3147 (LtnA2) and staphylococcin C55 (C55β). Homologous amino acids are indicated in bold, differences are indicated in red. E and F indicates evolutionary divergence (measured in the number of substitution events) between the unmodified lantibiotic propeptides (E) LtnA1 and C55α and (F) LtnA2 and C55β. (G) Visualization of homology modeling of LtnA1 (gold) and C55α (purple).

Lacticin 3147 and staphylococcin C55 are closely related two component lantibiotics. LtnA1 and C55α share 86% identity with a difference of only 4 amino acids in the unmodified propeptide whereas LtnA2 and C55β share less identity (55%) with highest homology spanning a 16 amino acid region at the C terminal end of the propeptides (Fig. 1). Thus, it is not surprising that the predicted structure of C55α very closely resembles that which has been established for LtnA1 (Fig. 1) [21]. In addition to the structural peptides the proposed modification and transport proteins exhibit significant homology. For example, the lacticin modification peptides lacticin LtnM1 and LtnM2 share 44.7% and 40.7% identity to their staphylococcin counterparts SacM1 and SacM2, respectively. In addition, the transport peptides lacticin LtnT and staphylococcin SacT are 48.9% identical, while lacticin LtnJ and staphylococcin SacJ (C55 orf45) share 47% identity. A clustal W alignment demonstrating the evolutionary divergence across the structural peptides of lacticin 3147 and staphylococcin C55 indicated that the number of substitution events between LtnA1 and C55α was considerably lower at 14.7 in comparison to 67 for LtnA2 and C55β (Fig. 1). In contrast, investigation of the evolutionary divergence between the modification peptides LtnM1 with SacM1 and LtnM2 with SacM2 indicated values of 93.8 and 104.5, respectively, while the transport peptides were slightly more conserved with a value of 81.6 for LtnT and SacT. These results indicate that the lanthionine containing peptides LtnA1 and C55α had a slow rate of divergence relative to the biosynthesis machinery and are highly conserved. Indeed, the amino acid identity and evolutionary divergence across both two component lantibiotics is indicative of a common ancestor.

### Purified preparation of staphylococcin C55 displays a broad spectrum of inhibition analogous to lacticin 3147

Initially, the range of inhibitory activity of *L. lactis *DPC3147 and *S. aureus *C55 against a number of indicator strains was examined (Table 1). The strain *S. aureus *C55 inhibited *L. lactis *strains and *M. luteus *but failed to inhibit *Enterococcus*, mastitic *S. aureus, Listeria*, *Bacillus*, *Leuconostoc*, *Pediococcus *or *Escherichia coli*. However, all of these strains with the exception of *Pediococcus *and the Gram-negative *E. coli *strain were inhibited by a concentrated preparation of the staphylococcin C55 peptides. The *L. lactis *DPC3147 culture inhibited all strains listed with the exception of the *S. aureus *strains and *E. coli*. However, when the concentrated supernatant of lacticin 3147 was assayed it was found to inhibit the full list of Gram-positive indicator strains used (Table 1). Overall, this demonstrates that both bacteriocins are active against a wide range of Gram-positive strains.

### Cross complementation between lacticin 3147 and staphylococcin C55 evident at a nanomolar range

**Figure 2:**
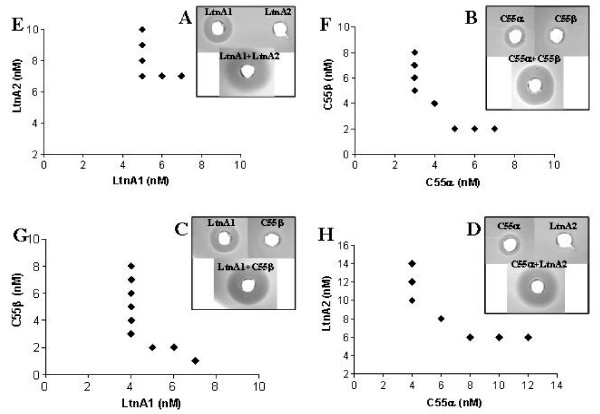
(A and B) Illustration of the requirement for synergistic activity of both structural components of the lantibiotics lacticin 3147 (LtnA1 and LtnA2) and staphylococcin C55 (C55α and C55β) for full activity of the lantibiotic. C and D illustrate cross complementation of the structural peptides of lacticin 3147 with those of staphylococcin C55. Isobologram graphs show nanomolar concentrations of purified peptides of lacticin 3147 with those of staphylococcin C55. Each point on the graph represents 50% growth inhibition of the indicator *L. lactis *HP. Isobolograms of (E) represent purified lacticin peptides LtnA1 and LtnA2 titrated against each other, (F) represent purified staphylococcin C55 peptides C55α and C55β titrated against each other, (G) represent purified lacticin LtnA1 peptide titrated against staphylococcin C55β and (H) represent purified staphylococcin C55α titrated against lacticin LtnA2 peptide.

The synergistic effect of staphylococcin peptides C55α and C55β and the lacticin 3147 peptides LtnA1 and LtnA2 against the indicator strain *L. lactis *HP is clearly shown by the well diffusion assay technique (Fig. 2A and 2B). When the lacticin 3147 and staphylococcin C55 peptides were cross combined, complementation was observed. For instance, when LtnA1 and C55β were combined, a zone of inhibition against the sensitive indicator strain *L. lactis *HP was evident (Fig. 2C). Indeed, the combination of C55α and LtnA2 also appeared to exhibit cross synergism to the level of activity presented by both parent strains (Fig. 2D). This cross complementation which was evident between the two strains was further examined by microtitre plate assays using purified preparations of both peptides and is presented as isobolograms (Fig. 2E–H). The MIC_50 _for the lacticin peptides was in the single-nanomolar range; i.e. 5 nM of LtnA1 titrated against 7 nM of LtnA2 inhibited the growth of the indicator *L. lactis *HP by 50%, as similarly reported by Morgan *et al*., (2005) (Fig. 2E). Likewise, combinations of staphylococcin C55 peptides also displayed inhibitory activity in the singular nanomolar range (4 nM) at a peptide stoichiometry of 1:1 (Fig. 2F). Similarly, hybrid pairs of the bacteriocins, LtnA1 with C55β (Fig. 2G) and C55α with LtnA2 (Fig. 2H), displayed a high specific activity of 4:3 nM and 8:6 nM, respectively. Overall, the level of inhibition across the four combinations was in the nanomolar range.

### Effect of swapping structural genes

**Figure 3:**
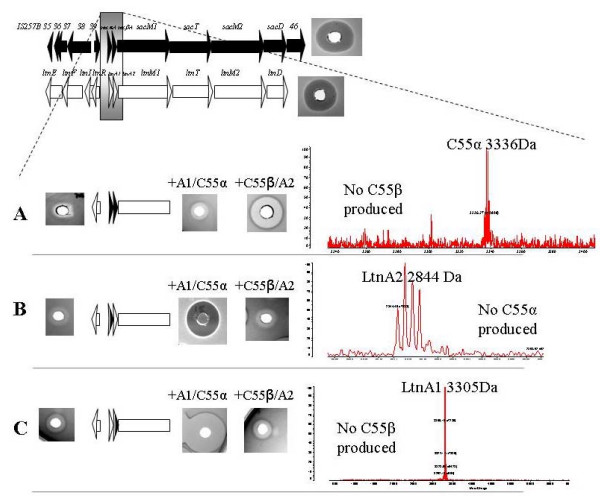
Genetic replacement study of combinations of the lacticin 3147 structural genes for the staphylococcin C55 structural genes on pMRC01. Genetic organization of *S. aureus *C55 pETB plasmid (gene cluster of staphylococcin C55) is represented by black arrows and *L. lactis *DPC3147 pMRC01 (gene cluster of lacticin 3147) is represented by white arrows. A. MG1363 pMRC01 with *ltnA1A2 *genes exactly replaced with staphylococcin C55 structural genes, *sacαAsacβA*, this construct is called Mutant A. B. MG1363 pMRC01 with *ltnA1 *structural gene exactly replaced by *sacαA *structural gene, this construct is called *ltn*A1/Sacα Hybrid. It also contains the wild type *ltn*A2 gene. C. MG1363 pMRC01 with *ltnA2 *structural gene exactly replaced by *sacβA *structural gene, this construct is called *ltn*A2/Sacβ Hybrid. It also contains the wild type *ltn*A1 gene. The well diffusion assay on the left of the constructs shows activity of a purified preparation (through a Varian C18 column) of the construct alone and the well diffusion assays on the right hand side shows activity after complementation with purified lacticin 3147 or staphylococcin C55 peptides. Mass spectrometry analysis of the constructs indicates the mass of the peptides produced. The scale of the y-axis of the mass spec. is A) 2800–3600 mass/charge, B) 2300–3700 mass/charge and C) 2700–4000 mass/charge.

The *ltnA1 *and *ltnA2 *genes in the large conjugative plasmid pMRC01 were precisely replaced with the staphylococcin C55 structural genes *sacαA *and *sacβA *using a strategy involving amplification and spliced overlap extension (SOEing) PCR [23] (Fig. 3A). This construct inhibited the indicator strain *L. lactis *HP, albeit the inhibitory activity was significantly reduced (Fig. 3A). The supernatant of mutant A was purified (solid phase extraction) and mass spectrometry analysis revealed that the structural peptide C55β was not produced whereas a mass corresponding to C55α was evident. When the purified HPLC fraction putatively containing C55α was combined with pure peptides of either LtnA2 or C55β the activity was greatly enhanced. This demonstrated that the modification and transport machinery of lacticin 3147 could modify and export the C55α peptide resulting in an extracellular mature active peptide. Notably, no complementation effect was seen when HPLC fractions were combined with either LtnA1 or C55α, supporting the mass spectrometry finding that C55β was not produced. It was thought possible that heterologous production of C55α and C55β could be enhanced through fusion of the corresponding LtnA1 and LtnA2 leaders, respectively, (which are more divergent than the corresponding propeptides) if it aided recognition of the peptides by the lacticin 3147 biosynthetic machinery.

Thus the leader of LtnA1 was fused by SOEing to the C55α propeptide segment of the structural gene generating an LtnA1/Sacα hybrid (Fig. 3B) and the leader of LtnA2 was fused to the propeptide segment of the structural gene of C55β generating an LtnA2/Sacβ hybrid (Fig. 3C). Purified preparations (solid phase extraction) from both hybrid bacteriocins produced a zone of inhibition against the indicator strain *L. lactis *HP, however, the activity was significantly reduced with respect to the parent strains. The reduced zone of inhibition was attributed to a concentrated level of one peptide alone, either LtnA2 (Fig. 3B) or LtnA1 (Fig. 3C). Following HPLC purification, neither hybrid peptide could be detected by mass spectrometry analysis. Notably, no complementation effect was seen when HPLC fractions of LtnA1/Sacα hybrid were combined with either LtnA2 or C55β (Fig. 3B) and similarly no complementation effect was seen when HPLC fractions of LtnA2/Sacβ hybrid were combined with either LtnA1 or C55α, supporting the mass spectrometry finding that the hybrid peptides were not produced. These results indicate that while the lacticin biosynthesis machinery can produce the active mature peptide C55α, it cannot produce either of the hybrid bacteriocins where Ltn leaders are used in attempt to enhance the generation of active mature processed C55 peptides.

### Site-directed mutagenesis reveal that residue L21 is essential for LtnA1 production

**Figure 4:**
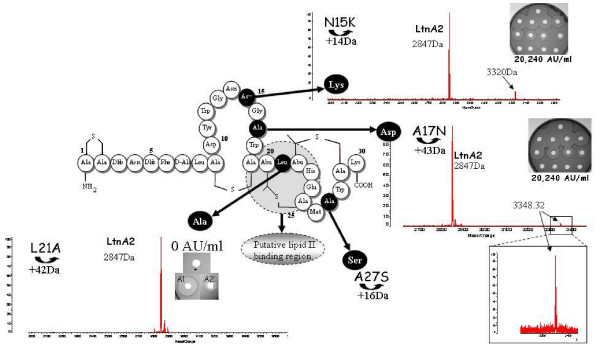
Site directed mutagenesis of the structural backbone of the lantibiotic peptide LtnA1. Mutants N15K, A17N and L21A are indicated by arrows. Well diffusion assays indicating the activity (AU/ml) of concentrated purified supernatants of the mutants (Varian C18 column) and mass spectrometry profiles accompany each mutant generated. The scale on the y-axis of the mass-spec is L21A: 2000–3700 mass/charge, A17N: 2700–3400 mass/charge, N15K: 2000–3600 mass/charge. The Mutant L21A has no activity (0 AU/ml), the + indicates that this mutant was combined with LtnA1 and LtnA2, and a complementation effect was evident with LtnA1. The mutant A27S could not be generated but represents the fourth amino acid difference between staphylococcin C55α and LtnA1. The shaded broken circle represents the putative lipid II binding region of LtnA1.

Site-directed mutagenesis of LtnA1 was carried out in order to assess the biological significance of changing any of the 4 amino acids in LtnA1 (N15, A17, L21 and A27) to their staphylococcin C55 counterparts. Several attempts to generate an A27S mutant failed. However, mutants N15K, A17N and L21A were introduced into the genes for the prepeptides. Therefore N15K, A17N amd L21A mutant peptides were successfully engineered. While concentrated preparations (solid phase extraction) of N15K and A17N supernatants retained high activity (20,240 AU/ml) the amino acid change of leucine to an alanine at position 21, which is in the putative lipid II binding region completely abolished the inhibitory capacity of the corresponding strain (0 AU/ml) (Fig. 4). Notably, when the purified preparation (solid phase extraction) of L21A was combined with LtnA1 peptide a complementation effect was evident confirming the mass spectrometry finding that LtnA2 was produced. On the other hand, when L21A was combined with LtnA2 peptide no complementation effect was evident supporting the mass spectrometry result that the LtnA1 mutant was not produced. In this case, a reduced zone of inhibition was evident; however, this was due to the presence of concentrated LtnA2 peptide alone. In fact it would appear that an LtnA1 derivative is not produced by this strain suggesting that the lacticin 3147 biosynthetic machinery could not tolerate this change. This is further evidence of a difference in the processing machinery of lacticin 3147 and staphylococcin C55, given that the SacM1 modification enzyme tolerates the presence of an alanine at position 21 in C55α.

## Conclusion

*L. lactis *DPC3147 and *S. aureus *C55 contain plasmids which encode highly similar two component lantibiotics. Indeed, evolutionary divergence and amino acid identity across the two lantibiotics confirm a common ancestor. The structural genes for the LtnA1 and C55α peptides have diverged to a much lesser extent than their associated modification and processing machinery, indicating a high degree of conservation between sequence, structure and function in these peptides, almost certainly imposed by the role of these peptides in mediating binding to a highly conserved target in lipid II and subsequently promoting interaction with their LtnA2/C55β partner peptides. It is interesting to note in this respect that the leader regions do diverge to the extent which would be predicted by the divergent modification genes. In contrast, far less conservation is required for the α-helical section of the LtnA2/C55β peptides, where it is envisaged that amphiphatic conservative changes would not negatively affect the overall function of the peptide in pore formation. In addition, the difference in inhibitory spectra for both strains is negligible, both inhibiting a wide range of Gram-positive bacteria. When combinations of purified peptides were assayed, for example, LtnA1 with C55β and C55α with LtnA2, they display a cross synergistic effect against the indicator strain *L. lactis *HP. In addition to this cross complementation between the lantibiotics, their specific activity was in the singular nanomolar range. This finding suggests that both lantibiotics use the lipid II target, despite differences in their lipid II binding pocket. It is not surprising that LtnA1 and C55α can be interchangeable as they share 86% identity while on the other hand, LtnA2 and C55β only share 55% identity. Nonetheless, when LtnA2 and C55β were exchanged for each other no loss of activity was observed. This suggests that the region responsible for peptide:peptide (and possibly peptide lipid II) interaction spans the 16 amino acid region at the C terminus of both peptides (81% identity), while the more permissive N-terminus is responsible for membrane insertion and pore formation. While these results are obviously of interest with respect to lacticin 3147 and staphylococcin C55 in particular, there are a number of broader implications. This is the first occasion upon which it has been demonstrated that the individual components of any sort of two-component bacteriocin, be they lantibiotics or non-lantibiotics, are interchangeable. It would also indicate that one could take advantage of the fact that functional domains are separated across two peptides. Theoretically A1/α and A2/β from different sources could be combined with a view to identifying pairs that have more potent activity against a particular target organism.

Interestingly, at a molecular level, the lacticin 3147-biosynthetic machinery was capable of producing the lanthionine containing C55α peptide when preceded by the staphylococcin C55α leader. To our knowledge this is the first occasion upon which an active (β-methyl) lanthionine-containing peptide has been produced from its own gene by biosynthetic machinery other than its own, without some manipulation of its leader region. Previously it was established that processed, albeit inactive, nisin could only be produced by the subtilin biosynthetic machinery if a nisin-subtilin chimera containing the subtilin leader was first generated [24]. It is thus surprising that when the C55 leader was precisely replaced with the lacticin LtnA1 leader (LtnA1/Sacα hybrid) production of C55α was eliminated rather than improved (Fig. 3B). It was previously established that processing of lacticin 481 by LctM occurred independently of the first 10 N-terminally located residues of the LctA leader [25]. Significantly, although the LtnA1 and C55α leaders are only 48% identical, they are 74% identical with respect to the 19 C-terminally located residues. This identity is likely to be responsible for successful biosynthesis and production by the lacticin 3147 machinery. It may also mean that one or more of the five residue differences within this region could explain the negative impact of the LtnA1 leader. Thus, in this instance at least, the leader appears to be specific to the peptide that it exports rather than to the biosynthetic machinery. With respect to C55β it is not surprising, given the relatively lower levels of homology with LtnA2 both within the structural and leader regions, that heterologous production was not successful.

Mature LtnA1 differs from C55α with respect to at least four residues. There is, however, some ambiguity with respect to the identity of the amino acid at position 7 of C55α. It may be that this residue is a D-alanine, as is the case for LtnA1, rather than the Dha reported previously [6]. This suggestion is based on the fact that amino acid analysis predicts the presence of three alanines in the mature form of C55α (data not shown) and that the C55 operon contains an LtnJ homolog [26]. With respect to the four confirmed differences site directed mutagenesis was employed to convert the individual amino acids of LtnA1 into its C55 counterpart where three out of the four mutants were successfully created. Interestingly, it was found that an exchange of leucine 21 for alanine abolished peptide production of the associated mutant peptide. It is noteworthy that a corresponding leucine is also found in a number of other lantibiotics within the same subgroup as LtnA1 i.e. mersacidin [27], actagardine [20], and plantaricin W [28]. Alignments of closely related globular peptides show that residues surrounding this leucine are highly conserved and in the case of mersacidin are found to be involved in lipid II binding [29]. In contrast, the site directed mutants of N15K and A17N tolerated change and the peptides were highly active. It may be that the presence of an alanine at position 21 of these peptides is only successful if complementary residues are located at positions 15, 17 and/or 27. It will be interesting to determine in the future whether it is possible to restore production/activity of the LtnA1-L21A mutant through the introduction of compensatory mutations.

We have thus established that because of their two component nature it is possible to combine the attributes of two lantibiotics such as lacticin 3147 and staphylococcin C55 in vitro to produce new antimicrobials. Should it prove possible to build on these successes a great number of two-component mixtures, limited only by the number of two-component lantibiotics present in nature, would be possible. It is very interesting that such remarkably similar systems ended up in dissimilar organisms.

## Methods

### Bacterial strains, culture conditions, inhibitory spectrum assays and cross complementation assays

Bacterial strains and plasmids used in this study are listed in Table 1 and Table 2. *L. lactis *subsp. *lactis *DPC3147, *L. lactis *subsp. *cremoris *HP and strains used in inhibitory spectra study (Table 1) were obtained from the culture collection at the Moorepark Food Research Centre, Teagasc, Fermoy, Co. Cork, Ireland. *S. aureus *C55, phage group II type71 strain [30], was obtained from Department of Microbiology, University of Otago. Lactococcal strains were grown at 30°C on M17 [31] supplemented with 0.5% w/v lactose (LM17) or glucose (GM17). *S. aureus *C55 was grown at 37°C on Tryptic Soy broth (Difco). Solid media was prepared by the addition of 1% agar. *L. lactis *DPC3147 and *S. aureus *C55 were grown in modified tryptone-yeast (TY) broth [32] for the purpose of peptide purification. All strains were stocked at -20°C in 40% glycerol. Bacteriocin activity of cultures, *L. lactis *DPC3147 and *S. aureus *C55, was assessed using deferred antagonism assay technique [33]. Briefly, 10 μl of overnight culture (10^8 ^cells per ml) was spotted on petri dishes containing 20 ml of solidified GM17 and allowed to grow overnight at 30°C or 37°C. The resultant growth was overlaid with molten agar containing 1 × 10^6 ^cells of indicator strain listed in Table 1. Inhibitory activity of the concentrated supernatant preparations from *L. lactis *and *S. aureus *was assessed using well diffusion assays described by Ryan *et al*., (1996). In this method, each indicator strain (10^6 ^cells per ml of an overnight culture), was seeded into molten agar (48°C). Following this, the concentrated supernatants of lacticin 3147 and staphylococcin C55 (50 μl) were dispensed into wells and the seeded plates were incubated at 30°C or 37°C. In addition, cross complementation assays of lacticin 3147 and staphylococcin C55 peptides was carried out by the well diffusion assay technique also, whereby molten LM17 agar was seeded with the indicator *L. lactis *HP and 25 μl of concentrated preparations of peptides were combined and assayed in wells.

### Bioinformatic analysis and homology modelling

Nucleotide sequence analysis was performed using the DNAStar software package (DNAStar, Madison, WI, U.S.A.). Sequence alignments were performed using the CLUSTAL method [34] of the Megalign program of the DNAStar software package. The 3D structure of LtnA1 which differs in sequence from C55α by just four amino acids was used as a backbone to predict a model structure for C55α. Homology modelling was performed using SWISS MODEL and PDB Viewer [35], a fully automated protein structure homology-modelling server. The amino acid sequence was mapped onto LtnA1. The resulting superimposition of C55α on LtnA1 was then visualised using the molecular viewer CHIMERA [36].

### Purification of lacticin (LtnA1 and LtnA2) and staphylococcin C55 (C55α and C55β) peptides

Overnight cultures of the producing strains, *L. lactis *DPC3147 and *S. aureus *C55 were inoculated into 2 L of modified TY broth (0.5% inoculum) and incubated overnight at 30°C and 37°C respectively. The cells were subsequently removed by centrifugation at 7,000 × g for 20 min. The resulting supernatant was applied to a column (3 by 23 cm) containing a 60 g bed of XAD-16 resin (Sigma-Aldrich Co. Ltd., Dorset, England) at a flow rate of 900 ml hour^-1 ^and the column then washed with 1 L of 40% aqueous ethanol. Inhibitory activity was subsequently eluted with 1 L of 70% propan-2-ol, pH 2 (adjusted to pH 2 with concentrated HCL). The propan-2-ol was removed by rotary evaporation and the resultant preparation was applied to a Varian C_18 _Bond Elut Column (Varian, Harbor City, CA) pre-equilibrated with methanol and water. The column was subsequently washed with 120 ml of 40% ethanol and inhibitory activity eluted with 100 ml of 70% propan-2-ol, pH 2. The bacteriocin preparation was concentrated to a volume of ~5 ml through the removal of propan-2-ol by rotary evaporation. Aliquots of 1 ml were then purified by solid phase extraction whereby aliquots were applied to a phenomenex C_18 _reverse phase (RP)-HPLC column (Phenomenex, Macctesfield, Cheshire, UK) previously equilibrated with 0.1% aqueous trifluoroacetic acid (TFA). The column was subsequently developed in a gradient of 27 to 53% propan-2-ol containing 0.1% TFA at a flow rate of 2.9 ml min^-1^. Fractions were collected and assayed for activity against the indicator strain *L. lactis *HP as described above. Each culture was assessed according to their HPLC profile and proposed fractions representing lacticin 3147 peptides LtnA1 and LtnA2 and staphylococcin C55 peptides C55α and C55β were subjected to mass analysis with a Shimadzu Biotech MALDI-TOF Mass Spectrometer (model AXIMA-CFR plus). In order to locate complementary activity during the purification protocol, 25 μl aliquots of putative LtnA1 fractions were cross-tested with isolated peptide LtnA2 and vice versa. Similarly, putative C55α fractions were combined with C55β to investigate complementation. Amino acid analysis of the lacticin 3147 peptides and staphylococcin C55 were performed in the Department of Molecular and Cell Biology, University of Aberdeen, Scotland.

### Specific activity determination and generation of isobologram graphs

Ninety six well microtitre plates were used to determine the minimum inhibitory concentration of 50% (MIC_50_) of *L. lactis *HP, using a combination of purified preparations of LtnA1, LtnA2, C55α, and C55β. Triplicate wells, containing 3 separate *L. lactis *HP cultures were included in each microtitre plate. The total volume in each well was 200 μl and experimental wells comprised broth (media), combinations of purified peptides LtnA1, LtnA2, C55α, C55β and 150 μl of a 1 in 10 dilution of overnight *L. lactis *HP cultures (diluted in growth media, L-M17). In addition, plates contained a number of blank wells (media only) and a number of control wells (untreated *L. lactis *HP, or *L. lactis *HP treated alone with LtnA1, LtnA2, C55α, or C55β) and plates were incubated at 30°C. The optical density at 600 nm (OD_600 _nm) was recorded at time 0 and 5 h (Anthos 2001, Anthos Labtec Instruments). Triplicate readings were averaged, and blanks (media only) were subtracted from these readings. A 50% growth inhibition was determined as half the final OD_600 _nm ± 0.05. Concentrations of combinations of peptides, which caused 50% growth inhibition of *L. lactis *HP, were plotted to generate an isobologram.

### Genetic replacement study

In order to exactly replace the lacticin 3147 structural genes *ltnA1 *and *ltnA2 *on pMRC01 with the staphylococcin C55 structural genes *sacαA *and *sacβA*, a spliced overlap extension (SOE) method was employed [23,37]. To generate this SOE product, primers SOEA, SOEB, SOE1, SOE2, SOE3 and SOED were designed (Table 3). Expand High fidelity (Roche diagnostics, East Sussex, UK) was used to amplify fragments for cloning. Two PCR products (P1 and P3), each 291 bp in length, were amplified from pMRC01 using primer pairs SOEA, SOEB and SOE3, SOED which amplify from either side of the *ltnA1A2 *genes. The third product (P2), 433 bp in length, was amplified from *S. aureus *C55 using primer pair SOE1 and SOE2, representing *sacαAsacβA*. The resulting products were gel extracted using the genecleanII kit (Qbiogene, Inc., Carlsbad, CA). Product P1 and P2 were mixed in a 1:1 ratio, and reamplified by PCR, with the SOEA and SOE2 primers, resulting in a new (P1+P2) fragment. P1+P2 was then mixed in a 1:1 ratio with P3 and reamplified by PCR with primers SOEA and SOED generating a spliced product (P1+P2+P3), whereby the staphylococcin C55 structural genes precisely replace the lacticin structural genes. This product was cloned in to pCR^®^2.1-TOPO^® ^vector (Invitrogen™ Life Technologies) and sequenced (Lark Technologies) and subsequently cloned into the erythromycin (Ery) resistant integration vector pORI280 generating the plasmid pORI-SOEA (Table 2). The plasmid pORI-SOEA was transformed into *E. coli *EC101 (RepA^+^) [38]. Blue colonies on Ery (150 μg ml^-1^) and 5-bromo-4-chloro-3-indolyl-β-D-galactosidase (X-Gal; 40 μg ml^-1^) on Luria-Bertani agar (Difco) were selected, and restriction digest was used to confirm the correct insert. The plasmid pORI-SOEA was then electroporated into competent *L. lactis *MG1363 cells [39,40] containing both pMRC01 and the temperature-sensitive chloramphenicol resistant (Cm^r^) RepA^+ ^helper plasmid pVE6007 [41]. Temperature induced curing of pVE6007 at 39°C along with the selective pressure of Ery resistance ensured the integration of pORI-SOEA into pMRC01. To excise pORI280, the Ery resistant and Cm sensitive colonies were passaged in 10 ml of GM17 broth without antibiotic selection at 39°C. After 12 consecutive transfers (0.1% inoculum), white colonies appeared indicating a second crossover event leading to the loss of pORI280 and the generation of Mutant A (Table 2). PCR was used to assess deletion-replacement of target genes, using check primers (Table 3) outside the SOE product in pMRC01 and internal to the C55 structural genes. The above method was also employed to generate MG1363 pMRC01 ltnA1leadersacαAΔltnA1 (LtnA1/Sacα Hybrid) and MG1363 pMRC01 ltnA2leadersacβAΔltnA2 (LtnA2/Sacβ Hybrid) which exactly replaced either the lacticin 3147 *ltnA1 *gene with staphylococcin C55 propeptide segment of the structural *sacαA *gene or the lacticin 3147 *ltnA2 *gene with staphylococcin C55 propeptide segment of the structural *sacβA *gene respectively, in pMRC01 (Table 2). The primers used to generate LtnA1/Sacα Hybrid were as follows; SOEA, SOE6, SOE7, SOE4, SOE5, and SOED (Table 3). The primers used to generate LtnA2/Sacβ Hybrid were SOEA, SOE8, SOE9, SOE2, SOE3, and SOED (Table 3).

### Residue specific mutagenesis

QuikChange XL site-directed mutagenesis (Stratagene) was used to incorporate changes into LtnA1 (changing it into its staphylococcin C55α counterpart) according to manufacturers instructions. Briefly, PCR of the template pORI280-ltnA1A2 generated by Cotter et al., (2005) was used to produce the DNA fragments incorporating the site directed changes encoded on the specifically designed oligonucleotide primers (Table 3). The products were digested with the enzyme *Dpn1 *and transformed into *E. coli *host EC101. In this way the following pORI280-LtnA1 derivatives were made: pORI-N15K, pORI-A17N and pORI-L21A (Table 2). PCR was used to screen for candidate *E. coli *EC101 transformants using check primers (Table 3). In all cases successful mutagenesis was confirmed by DNA sequencing. Mutants were electroporated into MG1363 pMRC01 pVE6007 and were incorporated into pMRC01 by double-crossover homologous recombination in place of the corresponding region (Table 2). DNA sequencing was used to confirm successful mutagenesis in the lactococcal hosts.

### Determination of bacteriocin activity

Bacteriocin activity was determined by the agar well diffusion assay as described by Parente and Hill [42]. Molten agar was seeded with an indicator strain and dispensed into petri dishes. Wells of approximately 4.6mm in diameter were bored in the agar and a 50 μl volume of a two fold serial dilution of a bacteriocin preparation was dispensed into each well.

## Authors' contributions

EBOC Primaryauthor and contributed to all experiments. PC reviewed, Site directed mutagenesis experiments, and intellectual contribution to the manuscript. POC Mass Spectrometry analysis, HPLC analysis and specific activity analysis. OOS performed the homology modelling. JRT intellectual input behind the cross complementation. RPR Principle investigator and intellectual input. CH Principle investigator and intellectual input. All authors have read and approved the final manuscript.

## Figures and Tables

**Table 1:** Inhibitory spectra of (A) *S. aureus *C55 and *L. lactis *DPC3147 culture and (B) *S. aureus *C55 and *L. lactis *DPC3147 supernatant purified from a C18 Varian column.

	**A**		**B**	
**Strain^a^**	**Culture^b^**		**Concentrated supernatant^b^**	
	*S. aureus *C55	*L. lactis *DPC3147	*S. aureus *C55	*L. lactis *DPC3147
*Enterococcus faecalis *DPC1145	NZ	+	+	++
*Enterococcus faecalis *DPC1143	NZ	+	+	++
*Enterococcus faecalis *DPC115	NZ	+	+	++
*Lactococcus lactis *DPC3147	+	NZ	+	+
*Lactococcus lactis *DPC481	+	++	++	++
*Lactococcus lactis *DPC497	+	++	++	++
*Lactococcus lactis *IL1403	+	+++	++	++
*Lactococcus lactis cremoris *HP	++	+++	++	+++
*Lactobacillus gasseri *DPC6123	+	++	++	+++
*Lactobacillus plantarium *DPC6124	NZ	+	+	++
*Lactobacillus paracasei *DPC6130	NZ	++	++	+++
*Staphylococcus aureus *C55	NZ	NZ	+	+
*Staphylococcus aureus *DPC5245	NZ	NZ	+	++
*Staphylococcus aureus *DPC5246	NZ	NZ	+	++
*Listeria monocytogenes *L028	NZ	+	+	++
*Listeria innocua *DPC3306	NZ	++	++	+++
*Bacillus subtilus*	NZ	+	++	++
*Micrococcus luteus*	+	++	++	+++
*Leuconostoc oenes*	NZ	++	++	++
*Pediococcus pentasaceus *DPC3541	NZ	+	NZ	++
*Pediococcus pentasaceus *DPC2445	NZ	+	NZ	+
*Escherichia coli *O157:H7	NZ	NZ	NZ	NZ

**a**. DPC, Moorepark Food Research Centre, Teagasc, Fermoy, Co. Cork, Ireland.

**b**. NZ, No zone; +, 1–5 mm; ++, 5–10 mm; +++, 10 mm and over

**Table 2:** Cloning strains and plasmids used in this study

**Strain or plasmid**	*Relevant characteristics*	*Reference*
**Strains**		

MG1363	Plasmid-free derivative of *L. lactis *712	Gasson, 1983
MG1614	Streptomycin resistant derivative of MG1363	Gasson, 1983
MG1363 (pMRC01)	MG1363 transconjugant harbouring pMRC01	Coakley *et al*., 1997
MG1363 (pVE6007)	MG1363 harbouring pVE6007, Cm^r^	Maguin *et al*., 1992
MG1363 pMRC01 pORI-SOEA pVE6007	MG1363 harbouring pMRC01, pVE6007 and pORI-SOEA	This study
MG1363 pMRC01 pORI-SOEB pVE6007	MG1363 harbouring pMRC01, pVE6007 and pORI-SOEB	This study
MG1363 pMRC01 pORI-SOEC pVE6007	MG1363 harbouring pMRC01, pVE6007 and pORI-SOEC	This study
MG1363 pMRC01 pORI-SOEA	MG1363 harbouring pORI-SOEA integrated into pMRC01	This study
MG1363 pMRC01 pORI-SOEB	MG1363 harbouring pORI-SOEB integrated into pMRC01	This study
MG1363 pMRC01 pORI-SOEC	MG1363 harbouring pORI-SOEC integrated into pMRC01	This study
Mutant A	MG1363 transconjugant harbouring pMRC01*sacαAsacβA*Δ*ltnA1A2*	This study
LtnA1/Sacα Hybrid	MG1363 transconjugant harbouring pMRC01*ltnA1leadersacαAΔltnA1 *(*sacαA *is fused to ltnA1 leader)	This study
LtnA2/Sacβ Hybrid	MG1363 transconjugant harbouring pMRC01*ltnA2leadersacβAΔltnA2 *(*sacβA *is fused to ltnA2 leader)	This study
*E. coli *EC101	*E. coli *JM101 with *repA *from pWV01 integrated into the chromosome	Law *et al*., 1995
*E. coli *EC101 pORI280	*E. coli *EC101 harbouring pORI280	Leenhouts *et al*., 1996
*E. coli *EC101 pORI-ltnA1A2	*E. coli *EC101 harbouring pORI280-ltnA1A2	Cotter *et al*., 2005
*E. coli *pORI-N15K	*E. coli *EC101 harbouring pORI-N15K	This study
*E. coli *pORI-A17N	*E. coli *EC101 harbouring pORI-A17N	This study
*E. coli *pORI-L21A	*E. coli *EC101 harbouring pORI-L21A	This study
MG1363 pMRC01 pVE6007 pORI-N15K	MG1363 harbouring pMRC01, pVE6007 and pORI-N15K	This study
MG1363 pMRC01 pVE6007 pORI-A17N	MG1363 harbouring pMRC01, pVE6007 and pORI-A17N	This study
MG1363 pMRC01 pVE6007 pORI-L21A	MG1363 harbouring pMRC01, pVE6007 and pORI-L21A	This study
MG1363 pMRC01 pORI-N15K	MG1363 harbouring pORI-N15K integrated into pMRC01	This study
MG1363 pMRC01 pORI-A17N	MG1363 harbouring pORI-A17N integrated into pMRC01	This study
MG1363 pMRC01 pORI-L21A	MG1363 harbouring pORI-L21A integrated into pMRC01	This study
LtnA1-N15K	MG1363 harbouring pMRC01 LtnA1-N15K	This study
LtnA1-A17N	MG1363 harbouring pMRC01 LtnA1-A17N	This study
LtnA1-L21A	MG1363 harbouring pMRC01 LtnA1-L21A	This study

**Plasmids**		

pORI280	RepA^- ^ori^+ ^β-galactosidase-positive Ery^r ^integration vector	Leenhouts *et al*., 1996
pORI-SOEA	*ltnsacαAsacβAltn *containing derivative of pORI280	This study
pORI-SOEB	*ltnA1leadersacαAltn *containing derivative of pORI280	This study
pORI-SOEC	*ltnA2leadersacβAltn *containing derivative of pORI280	This study
pVE6007	RepA^+^, Cm^r^, temperature sensitive derivative of pWV01	Maguin *et al*., 1992
pORI280-ltnA1A2	*ltnA1A2 *containing derivative of pORI280	Cotter *et al*., 2005
pORI-N15K	*ltnA1 *site directed mutant N15K derivative of pORI280	This study
pORI-A17N	*ltnA1 *site directed mutant A17N derivative of pORI280	This study
pORI-L21A	*ltnA1 *site directed mutant L21A derivative of pORI280	This study

**Table 3:** Primers used in this work

**Primer name**	**Primer sequence^a^**
**Genetic Replacement Study**	

SOEA	AAC TGC AGT TAT ATA TTT GCG GC
SOEB	GAT ATG AAC CTC CTT ATT
SOE1	AAT AAG GAG GTT CAT ATC ATG AAA AGT TCT TTT TTA
SOE2	TTC ATT TTT TAC TCC TAG CAA GCG CGC GTA CAA GC
SOE3	TAG GAG TAA AAA ATG AA 3
SOED	ACG AAT TCT CTT ACA GAG TT
SOE6	GAAGATGTATTTGGTGCGTGTAGTACAAACACTTTT
SOE7	CGCACCAAATACATCTTC
SOE4	ATTAATTAAAAATTTGTTATTAACTTATTTACACCAAGACAT
SOE5	GTTAATAACAAATTTTTAATTAAT
SOE8	GAGTCTCATGGAGGAGGAACACCTTTAGCCTTATTG
SOE9	TGTTCCTCCATGAGACTC

**Check primers genetic replacement study**	

CheckC55α1_for	AACACTTTTTCTTTGAGTG
CheckC55α_rev	GACATACATTCGTGAGTA
1CheckC55α_rev	ACAAGCTCCAAAAATATCA
CheckC55β2_for	ACCGGGGTGATAGGTTAT
1CheckC55β_for	TACTGATGATGAAGTATATGC
CheckC55β_rev	ATATAACCTATCACCCCG
Check*ltnR*_for	GAGATAGTACTCTGTAC
Check*ltnM1*_rev	GATTTATCTTCTCAATG

**Residue Specific Mutagenesis**	

N15K_for	CTCAGTGATTACTGGGGAAATTTAGGGGCTTGGTGTACACTCAC
N15K_rev	GTGAGTGTACACCAAGCCCCTAAATTTCCCCAGTAATCACTGAG
A17N_for	GATTACTGGGGAAATAACGGGAACTGGTGTACACTCACTCATG
A17N_rev	CATGAGTGAGTGTACACCAGTTCCCGTTATTTCCCCAGTAATC
L21A_for	AATAACGGGGGGTGGTGTACAGCAACTCATGAATGTATGGCTTG
L21A_rev	CAAGCCATACATTCATGAGTTGCTGTACACCACCCCCCGTTATT
A27S_for	CACTCACTCATGAATGTATGTCGTGGTGTAAATAAGTTAATAAC
A27S_rev	GTTATTAACAATTTTACACCACGACTAACATTCATGAGTGAGTG

**Check primers residue specific mutagenesis**	

CheckN15K_for	GATTACTGGGGAAATTTA
CheckA17N_for	TGGGGAAATAACGGGAAC
CheckL21A_for	GGGGGGTGGTGTACAGCA
CheckA27S_for	ACTCATGAATGTATGTCG
CheckSDM_rev	TGTCGAATTCATTTCTGGAAAAAC

**a**. Primer sequences are given in the 5' to 3' direction
